# A Genome-Wide Profiling of Glioma Patients with an IDH1 Mutation Using the Catalogue of Somatic Mutations in Cancer Database

**DOI:** 10.3390/cancers13174299

**Published:** 2021-08-26

**Authors:** Amrit L. Pappula, Shayaan Rasheed, Golrokh Mirzaei, Ruben C. Petreaca, Renee A. Bouley

**Affiliations:** 1 Department of Electrical and Computer Engineering, The Ohio State University, Columbus, OH 43210, USA; pappula.1@buckeyemail.osu.edu; 2 Department of Microbiology, The Ohio State University, Columbus, OH 43210, USA; Rasheed.28@osu.edu; 3 Department of Computer Science and Engineering, The Ohio State University at Marion, Marion, OH 43302, USA; mirzaei.4@osu.edu; 4 Department of Molecular Genetics, The Ohio State University at Marion, Marion, OH 43302, USA; 5 Cancer Biology Program, The Ohio State University, Columbus, OH 43210, USA; 6 Department of Chemistry and Biochemistry, The Ohio State University at Marion, Marion, OH 43302, USA

**Keywords:** glioma, isocitrate dehydrogenase, IDH1, CIC, ATRX, somatic mutations, gene expression, TCGA, COSMIC

## Abstract

**Simple Summary:**

Glioma patients that present a somatic mutation in the isocitrate dehydrogenase 1 (IDH1) gene have a significantly better prognosis and overall survival than patients with the wild-type genotype. An IDH1 mutation is hypothesized to occur early during cellular transformation and leads to further genetic instability. A genome-wide profiling of glioma patients in the Catalogue of Somatic Mutations in Cancer (COSMIC) database was performed to classify the genetic differences in IDH1-mutant versus IDH1-wildtype patients. This classification will aid in a better understanding of how this specific mutation influences the genetic make-up of glioma and the resulting prognosis. Key differences in co-mutation and gene expression levels were identified that correlate with an improved prognosis.

**Abstract:**

Gliomas are differentiated into two major disease subtypes, astrocytoma or oligodendroglioma, which are then characterized as either IDH (isocitrate dehydrogenase)-wild type or IDH-mutant due to the dramatic differences in prognosis and overall survival. Here, we investigated the genetic background of IDH1-mutant gliomas using the Catalogue of Somatic Mutations in Cancer (COSMIC) database. In astrocytoma patients, we found that IDH1 is often co-mutated with TP53, ATRX, AMBRA1, PREX1, and NOTCH1, but not CHEK2, EGFR, PTEN, or the zinc finger transcription factor ZNF429. The majority of the mutations observed in these genes were further confirmed to be either drivers or pathogenic by the Cancer-Related Analysis of Variants Toolkit (CRAVAT). Gene expression analysis showed down-regulation of DRG2 and MSN expression, both of which promote cell proliferation and invasion. There was also significant over-expression of genes such as NDRG3 and KCNB1 in IDH1-mutant astrocytoma patients. We conclude that IDH1-mutant glioma is characterized by significant genetic changes that could contribute to a better prognosis in glioma patients.

## 1. Introduction

Gliomas are the most common form of malignant primary brain cancers, and for high-grade gliomas, such as glioblastoma, the prognosis is very poor, with patient survival of less than 2 years [1,2,3]. Gliomas, like other cancers, are associated with a large accumulation of somatic mutations and alterations in gene expression that contribute to their specific phenotype [4,5]. Mutations in the isocitrate dehydrogenase (IDH) family, notably IDH1 and IDH2, have been identified in several cancers such as grade II and III gliomas and acute myeloid leukemia (AML) [6,7,8]. Specifically, IDH mutation has become a hallmark of grade II and III gliomas and is also associated with a favorable prognosis [9,10,11,12,13]. IDH-mutant gliomas are split into either astrocytoma or oligodendroglioma subtypes, which have unique genetic and molecular profiles [14]. The presence of this mutation has such a dramatic effect on long-term survival that astrocytomas are then often further classified as either IDH-mutant or IDH-wildtype [14,15,16,17,18,19]. IDH2 can also be mutated, analogous to what is observed for IDH1, but it is generally observed with a lower frequency. In gliomas, heterozygous mutations in IDH1 occur exclusively at residue R132, which is usually changed to a histidine [6,20]. This residue is located within the active site of the enzyme and impairs its ability to convert isocitrate to α-ketoglutarate [21]. More importantly, this R132H mutation confers a novel gain of function that allows the mutant enzyme to convert α-ketoglutarate to D-2-hydroxyglutarate [22], which is a known oncometabolite that has been shown to inhibit histone demethylases and activate mTOR signaling [23,24,25,26,27,28,29].

There have been conflicting data regarding the driver or oncogenic potential of IDH1/2 mutations [8,29,30,31,32]. Recent studies have shown that mutation of IDH occurs very early in cancer progression and may drive genetic instability and mutations of other known oncogenes [33,34,35,36,37,38]. A thorough analysis of the genetic signatures of glioma cancer cells harboring an IDH mutation is needed to understand how this mutation influences cancer progression. Since an IDH1 mutation is well known to be associated with a better overall survival and response to chemotherapy treatment, such as temozolomide, compared to IDH1-wildtype glioma, this understanding could help to identify biological targets that could be exploited to improve patient outcomes. Generally, glioblastoma, the most aggressive form of glioma, does not harbor an IDH1 mutation and requires establishment of new treatments and biological targets [6,39]. The development of unique molecular signatures of glioma with or without an IDH mutation will help to shed light on possible targets that could be exploited in the treatment of glioblastoma. This study aims to specifically identify co-occurring mutations and gene expression patterns in IDH1-mutant glioma using a genome-wide approach, with the aim to improve glioma genetic profiling and understand how an IDH1 mutation influences this [40,41,42,43,44,45,46,47,48,49,50].

The Catalogue of Somatic Mutations in Cancer (COSMIC) is a repository of cancer mutation data from various studies including The Cancer Genome Atlas (TCGA), the International Cancer Genome Consortium (ICGC), and various other independent studies [51,52,53]. We analyzed the COSMIC database to first look at the tissue distribution of both IDH1 and IDH2 mutations and then exhaustively catalogue the various IDH1/2 mutations that occur in various tissues. This showed a clear majority of IDH1 mutations in the central nervous system as expected and a majority of IDH2 mutations in the hematopoietic and lymphoid systems. Since only a small percentage of IDH2 mutations are in the central nervous system, we chose to focus the rest of our analysis only on IDH1 mutations. All the IDH1 mutations in the central nervous system occurred exclusively at residue R132. We then utilized the COSMIC database to uncover mutations that frequently co-occur in samples with mutated IDH1. We found that astrocytoma patients with an IDH1 mutation have a unique molecular profile compared to IDH1-wildtype [54].

## 2. Materials and Methods

### 2.1. COSMIC Database

Version 94 of the COSMIC database was used for all data analysis.

### 2.2. Tissue Distributions of IDH1/2 Mutations

**Figure 1:**
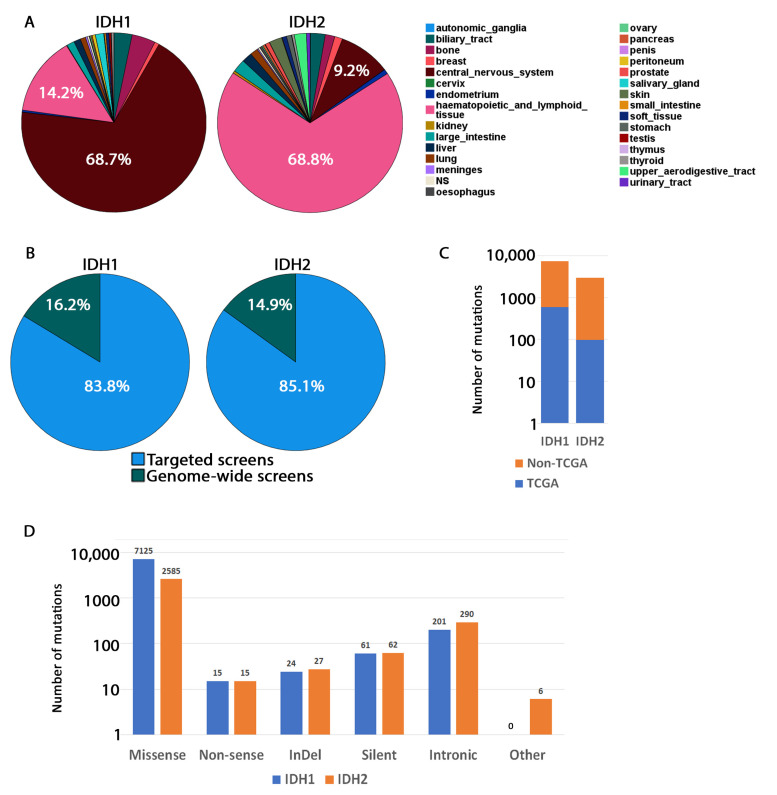
Mutations distribution in IDH1 and IDH2 in samples reported on COSMIC. (**A**) Pie charts showing tissue distribution of IDH1 and IDH2 mutations in all samples reported on COSMIC. Both targeted and genome-wide screens are included. (**B**) Percent distribution of samples from targeted and genome-wide screens. (**C**) Origin of genome-wide screens only. The majority of screens are from TCGA. Others are compiled from various published studies indexed on PubMed. (**D**) Number of IDH1 and IDH2 somatic mutations by type of mutation.

IDH1 and IDH2 mutation files were independently downloaded from COSMIC. COSMIC reports both genome-wide and targeted screens studies and the mutation profiles in Figure 1 includes both. Subsequent analyses were carried out with genome-wide screens only. COSMIC Mutation Data was used for the combined targeted and genome-wide screen data, which was filtered by gene name for IDH1. COSMIC Mutation Data (Genome Screens) was used for the genome-wide screens only data, which was filtered by gene name for IDH1.

### 2.3. Occurrence of Mutations in Glioma with an IDH1 Mutation

COSMIC Mutation Data (Genome Screens) was used to obtain the sample names for the IDH1 dataset, which was filtered by tissue (central_nervous_system) and gene (IDH1). The glioma control set was obtained by filtering only by tissue. The datasets were then further filtered by glioma as the histology site and primary tumor origin. For the IDH1 dataset, the sample names obtained were matched in the glioma control set and the data extracted. For the coding mutations, mutations in introns were removed to ensure all mutations were coding and alternative transcripts were also removed from the datasets. Noncoding mutations were split by promoter, terminator, and intronic mutations. Finally, each dataset was sorted by histology subtype as either astrocytoma or oligodendroglioma. For each gene mutated in the datasets, the number of individual patients was counted using the unique sample name identifiers. The fraction of patients with a mutation for each gene was calculated by dividing their number by the total number of unique patients in the datasets.

### 2.4. CRAVAT Analysis

The online CRAVAT tool (https://www.cravat.us/CRAVAT) was used for this analysis (accessed on 29 July 2021). We used both VEST and CHASM-3.1 analysis programs. For CHASM, we chose “Brain-lower-grade-glioma” as the disease type. Only scores with a probability value and false discovery rate (FDR) below 0.05 were considered statistically significant.

### 2.5. Gene Expression Levels in Glioma with an IDH1 Mutation

COSMIC Gene Expression was used to download Z score data filtered by tissue (central_nervous_system). Computational analysis was performed in Python 3 based on an object-oriented framework. The TCGA sample names were compiled from the datasets generated for the mutational analysis, and the data were extracted to generate the IDH1-mutant (*n* = 24) and IDH1-wildtype astrocytoma (*n* = 588) datasets. The process includes different algorithms/modules, including sorting algorithms to sort data based on sample names, search algorithms to find overlapping sample names, extraction algorithms to extract the genes/sample names, performing statistical *t* tests on Z scores, and final gene extraction based on specified criteria (*p* < 0.05, Z score > 2 or Z score < −2). A two-sided *t* test was performed using the Scipy Python library to compute the test for the means of the two independent samples of scores to measure whether the average (expected) value differed significantly across samples. We assumed that the populations had identical variances. A search algorithm was developed to locate the significant genes with *p* < 0.05. Average Z scores were calculated for both IDH groups. The overall diagram of the computational analysis for astrocytoma IDH1-mutant and -wildtype is shown in Figure S1.

## 3. Results and Discussion

### 3.1. Tissue Distribution of IDH1/2 Mutations

To understand the tissue distribution of IDH1/2 somatic mutations, targeted and genome-wide screens were queried from the COSMIC database. IDH1 somatic mutations were found primarily in the central nervous system (68.7%) and to a lesser extent in hematopoietic and lymphoid systems (14.2%), which agrees with the types of cancers IDH1 mutations have been associated with [7]. Whereas IDH2 mutations were found primarily in hematopoietic and lymphoid systems (68.8%) and to a lesser extent in the central nervous system (9.2%) (Figure 1A,B) [6,55], all other tissues had less than 10% of IDH1 or IDH2 mutations. COSMIC presents data from both genome-wide screens and targeted screens with most data being collected from targeted screens (Figure 1B). However, the mutation distribution of targeted screens mirrors that of genome-wide screens (Figure S2). A fraction of mutations was from the TCGA study (584 out of 11,490 for IDH1 and 99 out of 2930 for IDH2), while the others were from other studies (Figure 1C).

Next, we catalogued the type of mutations that occur in IDH (Figure 1D and Figure S2). For both IDH1 and IDH2, missense mutations were observed with the highest frequency followed by intronic and silent mutations. The R132 residue was mutated with the highest frequency in IDH1 for both targeted and genome-wide screens in agreement with previous studies [56]. In IDH2, R172 (analogous to R132 in IDH1) is mutated most frequently in the genome-wide only screen and is the second-most frequently mutated in both targeted and genome-wide screens [57,58]. R140 was mutated most frequently in the combined targeted and genome-wide screens and second-most frequently mutated in the genome-wide only screen. All these residues are located within the active site of the enzymes and directly impact catalytic activity.

Finally, the missense, nonsense, and silent amino acid mutations were classified by tissue type (Figure S3). As previously reported, the IDH1 R132 mutation was found primarily in the central nervous system, hematopoietic and lymphoid systems, bone, and biliary track, whereas other tissues showed a multitude of mutations [7,56,59]. The IDH2 R140 mutation was found primarily in the hematopoietic and lymphoid systems and notably was not found in the central nervous system [59,60]. The R172 mutation is represented in the central nervous system, hematopoietic and lymphoid systems, bone, and biliary tract, analogous to what is observed for the IDH1 R132 mutations [61]. It is also important to note that in the central nervous system, IDH1 R132 was mutated primarily to histidine (H), while other substitutions were less likely [10].

To understand why arginine is most often mutated to histidine in IDH1, we investigated the potential single nucleotide changes for the CGT codon which occurs at residue 132. It is an established fact that there is a codon bias usage in organisms including humans [62,63,64,65]. Remarkably, the CGT codon is the rarest arginine codon (8% frequency) (Figure S4). One reasonable proposed hypothesis suggests that codon bias drives both synonymous and nonsynonymous mutation rates; that is, mutation is driven towards higher frequency codon usage [66]. However, this hypothesis has subsequently been reinterpreted because such observations were not made in other studies [67,68,69]. Evolutionary mutation bias is driven by a complex interplay between permitted amino acid changes (highest frequency for one base pair substitution) and purifying selection that will eliminate substitutions to amino acids that drastically affect the function of the protein [70]. Cancer mutations are subject to the same forces and are also non-random [71]. Remarkably, arginine is the most mutated amino acid in cancer cells, but it has an almost equal probability to be changed to either histidine or cysteine (Figure S4). Histidine, like arginine, is a polar basic amino acid while cysteine is polar but weakly acidic (Figure S4D) [71]. Arg to Gln mutations are also very likely in cancer but not possible from the CGT codon.

### 3.2. Occurrence of Mutations in Glioma with an IDH1 Mutation

**Figure 2:**
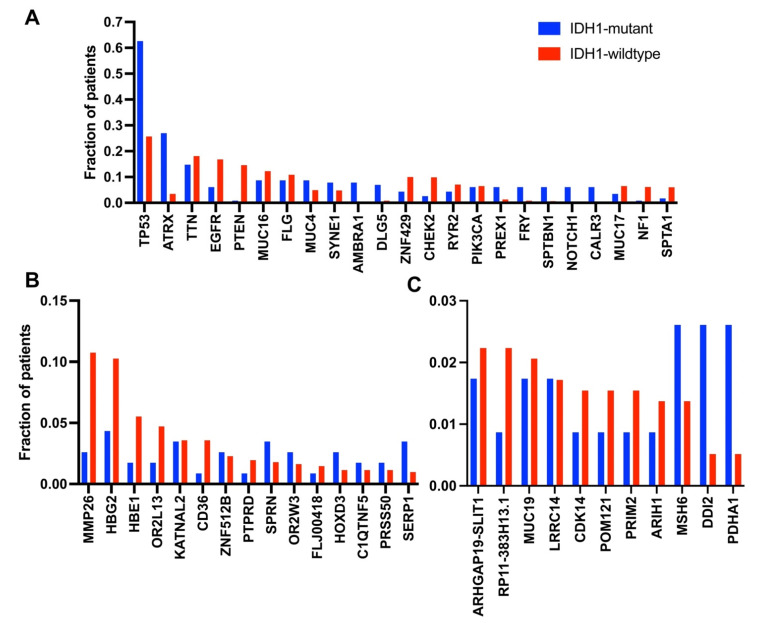
Frequency of mutations in IDH1-mutant and -wildtype astrocytoma patient samples. The number of individual patients displaying a mutation in each gene was counted, and the percentage was calculated from the total number of patients in the dataset. Mutations were split by (**A**) coding, (**B**) promoter, and (**C**) terminator. Mutation patient frequencies are shown for each analysis in Supplemental Tables S1–S3, respectively. Intronic mutations are not shown but are included in Supplemental Table S4. Functions and full names of each gene are included in Supplemental Table S5.

The COSMIC database was used to determine what other genes are co-mutated with IDH1 in astrocytoma patients. The number of individual patients displaying a coding mutation in a specific gene was determined for IDH1-mutant and IDH1-wildtype patients, and then the fraction of patients with a specific mutation was compared (Figure 2A, Table S1). The top two genes that displayed a higher frequency of mutations in astrocytoma patients with an IDH1 mutation were TP53 (63%) and ATRX (27%), which has been well documented [4,72,73,74]. Mutations in TP53 are well known to be associated with a variety of cancers and have been associated with both IDH1 and ATRX mutations in glioma [33,75,76,77]. An association between IDH1 mutations and reduced ATRX expression, which increases telomere length, has also been shown in grade II gliomas [74,78,79]. Other genes that were preferentially co-mutated with IDH1 were AMBRA1, DLG5, PREX1, FRY, SPTBN1, NOTCH1, and CALR3. AMBRA1 is an autophagy protein that regulates gene expression and induces autophagy [80]. In addition, AMBRA1 has also been associated with resistance to several chemotherapy drugs [78,79]. DLG5 plays a role in cell migration, adhesion, and proliferation, and mutations in DLG5 have been associated with bladder cancer [81,82]. PREX1 is a Rho GTPase and is thus involved in regulating cellular functions such as cell migration and adhesion [83,84]. Mutations and overexpression of PREX1 have been linked to several cancers including glioblastoma. SPTBN1 appears to play an anticancer role and negatively affects cell migration [85,86]. Changes in expression and mutations in NOTCH1 have been linked to a variety of cancers, including glioma [87,88,89,90,91].

An increased frequency of mutations in EGFR, PTEN, ZNF429, and CHEK2 was instead primarily found in IDH1-wildtype patients. Mutations in EGFR are commonly found in glioblastoma and promote astrogenesis [92]. Mutations or deletions of PTEN have also been identified in glioblastoma and affect the same signaling network as EGFR mutations [4]. CHEK2 is a cell-cycle checkpoint gene and has been shown to phosphorylate both BRCA1 and P53 [93]. Cancer-associated mutations of CHEK2 are generally inactivating, which impairs its ability to suppress cell proliferation [94]. Hereditary mutations in CHEK2 have also been shown to predispose patients to multiple cancer types [95]. In general, mutations in CHEK2 are associated with poor prognosis and reduced patient survival [96,97]. Thus, the decreased frequency of CHEK2 mutations in IDH1-mutant patients improves our understanding of why the IDH1-mutation phenotype is associated with an improved prognosis over IDH1-wildtype.

We also performed the analogous analysis of promoter and terminator mutations in either IDH1-mutant or -wildtype astrocytoma patients (Figure 2B,C, Tables S2 and S3). Notable differences in promoter mutation frequency were observed in MMP26, a matrix metalloproteinase, whose expression has been linked to tumor invasion [98,99,100,101]. A higher frequency of promoter mutations in SERP1 was observed in IDH1-mutant patients, which has been identified as a cancer biomarker [102,103]. We also analyzed the intronic mutations (Table S4), which showed many mutations in the protocadherin gene cluster in both IDH1-mutant and wildtype astrocytoma patients. The protocadherins are highly expressed in the brain and likely critical for neuronal connections [104,105]. Alterations in methylation and expression of these protocadherin gene clusters has been linked to several neurological disorders [106,107].

Oligodendroglioma patient data were also compiled and split by IDH1 mutation status. We observed that IDH1 mutation is strongly correlated with oligodendroglioma, as it was observed in 75 out of 85 patients. This means that our IDH1-wildtype group was extremely small (*n* = 10), which makes it difficult to draw significant conclusions from these data. The gene that showed the highest frequency of mutation was CIC, which has been previously reported to be associated with oligodendrogliomas, low grade gliomas, and an activated RAS-MAPK signaling pathway (Figure S5, Table S6). PIK3CA and NOTCH1 mutations were also strongly correlated with IDH1 mutational status. We also analyzed noncoding mutations in oligodendroglioma patients and observed similar genes identified in astrocytoma except for a lack of promoter mutation in MMP26 (Figure S5, Tables S7–S9).

**Figure 3:**
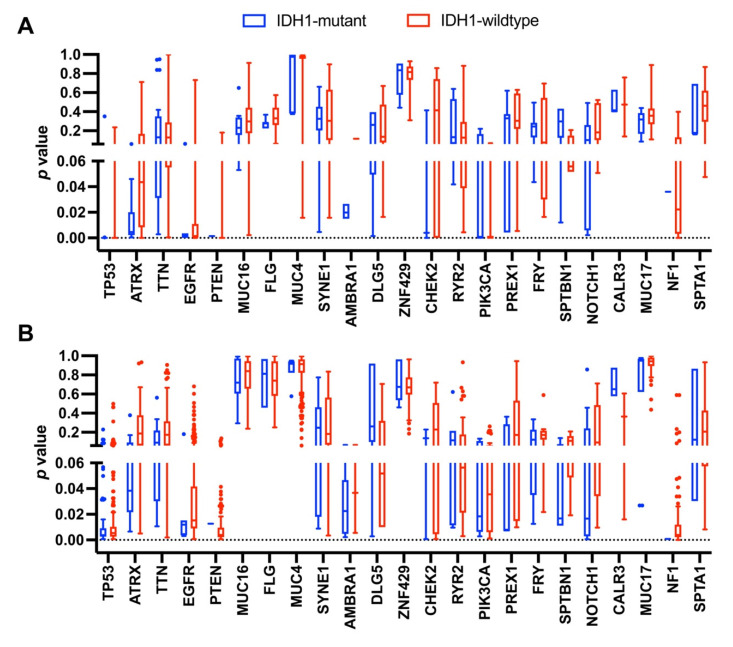
CRAVAT analysis of mutations observed in IDH1-mutant and wildtype astrocytoma patients. (**A**) CHASM and (**B**) VEST scoring was performed to calculate *p* values of each coding mutation observed in the genes displayed in Figure 2A to determine if mutations were drivers or pathogenic, respectively (*p* < 0.05). The *p* values for genes analyzed are shown as Tukey box plots with all outlier data points shown. All data are presented in Supplemental Tables S10 and S11.

Finally, we used the Cancer-Related Analysis of Variants Toolkit (CRAVAT) to determine if the coding mutations observed in astrocytoma IDH1-mutant patients were pathogenic or driver mutations [108]. Variants were scored using both CHASM (Cancer-Specific High-throughput Annotation of Somatic Mutations) and VEST (Variant Effect Scoring Tool) to determine *p* values (Figure 3, Tables S10 and S11). This showed that the majority of TP53 mutations had *p* values less than 0.05 for both scoring functions and are thus predicted to be drivers as well as pathogenic. The majority of ATRX missense mutations in the IDH1-mutant patients were also found to be drivers by CHASM and only found in the central nervous system. Many of ATRX variants were nonsense mutations, but only half of these were predicted to be pathogenic by VEST. Interestingly, the average *p* value for ATRX mutations in IDH1-wildtype patients was greater than observed for the IDH1-mutant patients, suggesting that altering the functions of both ATRX and IDH1 inhibits cellular transformation or cancer progression (Figure 3). The only other genes that, on average, had either driver and/or pathogenic variants were EGFR, PTEN, AMBRA1, PIK3CA, SPTBN1, NOTCH1, and NF1. However, there was only one variant that could be scored for PTEN and NF1, which in each case was predicted to be a driver. We also analyzed the IDH1 missense mutations observed (R132H, R132C, and R132G) to determine their *p* values, which were all below 0.05 for both CHASM and VEST (Table S10).

### 3.3. Gene Expression Levels in Glioma with an IDH1 Mutation

**Figure 4:**
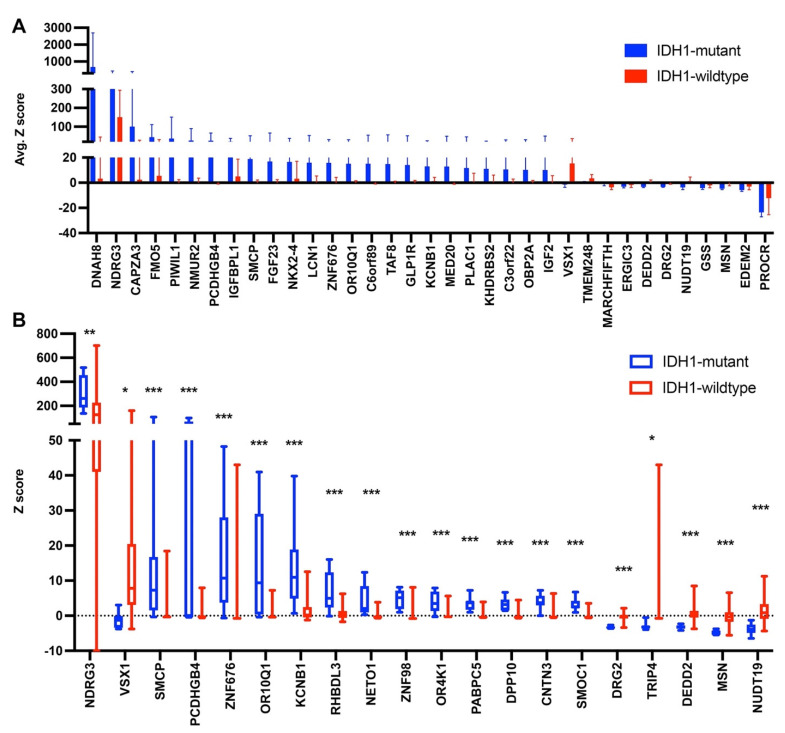
Gene expression levels in astrocytoma with or without an IDH1 mutation. (**A**) Average Z scores and standard deviations are plotted for genes that show statistically different expression between the two glioma subtypes (*p* < 0.05) and average Z scores > 10.0 or <−2.0. (**B**) Z score distributions for select genes are shown as box plots showing the 25th to 75th percentile as the box, the median as a central line, and the minimum to maximum values as whiskers. A two-tailed *t* test was performed to determine statistically different expressions between the two subtypes for each gene (* *p* < 0.05, ** *p* < 0.01, *** *p* < 0.001). All data are shown in Supplemental Table S12.

A computational analysis using Python was used to analyze expression data for IDH1-mutant astrocytoma versus IDH1-wildtype astrocytoma. Unfortunately, there were no expression data available for the corresponding analysis of oligodendroglioma. Expression data were compiled for each relevant patient, and the datasets were compared to identify genes that showed statistically different expressions using a two-tailed *t* test (Table S12). Following this, Z score averages and distributions were plotted for select genes that had statistically significant differences and on average were over- or under-expressed (Figure 4). The Z scores demonstrate the standard deviations from the mean for all patients. Thus, a Z score that is greater than 2 is significantly higher than normal, and a Z score below -2 is significantly lower than normal.

Sorting by the highest average Z scores showed DNAH8 as the gene with the highest expression in the IDH1-mutant patients (Figure 4A); however, only one patient out of eight showed high expression of this gene. Thus, this gene is most likely not significantly correlated with IDH1 mutation. The gene NDRG3 showed the highest expression in IDH1-wildtype astrocytoma and the second highest expression in IDH1-mutant astrocytoma. While both IDH1 groups showed high expression of NDRG3, the difference was found to be statistically significant (Figure 4B). NDRG3 (N-Myc Downstream-Regulated Gene 3) has been shown to be associated with a poor prognosis of several cancer types and control of hypoxia-inducible factors [109,110,111,112]. Over one hundred genes were found to be over-expressed in astrocytoma patients with an IDH1-mutation and show a statistically significant difference in expression from the IDH1-wildtype patients (Table S12), of which 24 are shown in Figure 4A and 13 genes with the lowest *p* values are shown in Figure 4B. A few of those genes are discussed in more detail here. VSX1 (visual system homeobox gene) mutations have been associated with keratoconus, which is a disease affecting the cornea, but this gene has not been linked to cancer [113]. PCDHGB4 is part of the protocadherin gamma subfamily, which are highly expressed in the brain and is likely critical for neuronal connections [109,110,114]. Alternations in methylation and expression of these protocadherin gene clusters has been linked to cancer and several neurological disorders [106,107]. ZNF676 is a zinc-finger protein that has been linked to the regulation of telomere homeostasis [115,116]. OR10Q1 encodes for an olfactory receptor that has so far not been studied [117]. KCNB1 is a potassium voltage-gated channel that is highly expressed in the brain and has been identified as a biomarker for colorectal cancer and associated with a favorable prognosis in glioma patients [114,118,119]. Specifically, it was shown that KCNB1 regulates autophagy through the ERK signaling pathway and acts as a tumor suppressor [114].

In addition, there were several genes identified that were significantly under-expressed in IDH1-mutant astrocytoma compared to IDH1-wildtype. DRG2, developmentally regulated GTP-binding protein 2, has been shown to promote tumor growth and metastasis [120,121,122]. Depletion or inhibition of DRG2 was shown to promote survival in mice [122]. TRIP4, which is a thyroid hormone receptor interactor, also promotes cell proliferation and migration. On the other hand, DEDD2 (death effector domain containing 2) associates with caspases to signal cell death and initiate apoptosis [123,124,125]. MSN (moesin) is important for cell movement, and is associated with cell proliferation and invasion in glioblastoma as well as other cancers [126,127,128,129]. Moesin expression has already been correlated with higher-grade astrocytoma and lower overall survival, but has not been correlated with IDH1 mutation status [126].

## 4. Conclusions

Our analysis identified signature genetic changes in IDH1-mutant and -wildtype astrocytoma that aid in understanding the differences in overall survival and prognosis of these cancers. IDH1-mutant astrocytoma demonstrates a phenotype that shows increased mutations in TP53, ATRX, AMBRA1, DLG5, PREX1, and NOTCH1. Mutations in CHECK2, EGFR, PTEN, RYR2, and NF1 are instead associated with an IDH1-wildtype astrocytoma. TP53 is one the most frequently mutated in both and thus would not serve as a reliable tool for understanding the differences in these subtypes. Our analysis of mRNA expression levels showed that two genes that promote cell proliferation and invasion, DRG2 and MSN, were under-expressed in IDH1-mutant astrocytoma compared to normal tissue and IDH1-wildtype patients. There was also significant over-expression of genes such as NDRG3 and KCNB1 in IDH1-mutant astrocytoma patients.

## Data Availability

Data were obtained from the COSMIC database, which is freely available for non-commercial users. The analyzed data presented in this study are available in Supplementary Tables S1–S12.

## Supplementary Materials

The following are available online at https://www.mdpi.com/article/10.3390/cancers13174299/s1, Figure S1: The diagram for computational analysis of astrocytoma IDH1-mutant and -wildtype groups, Figure S2: IDH1 and IDH2 mutation distribution in all sequenced samples, Figure S3: IDH1 and IDH2 tissue distribution of mutations in genome wide screens, Figure S4: Mutation probability of the CGT codon that codes for arginine, Figure S5: Frequency of mutations in IDH1-mutant and -wildtype oligodendroglioma patient samples, Table S1: Coding mutation frequency in IDH1-mutant or -wildtype astrocytoma patients, Table S2: Promoter mutation frequency in IDH1-mutant or -wildtype astrocytoma patients, Table S3: Terminator mutation frequency in IDH1-mutant or -wildtype astrocytoma patients, Table S4: Intronic mutation frequency in IDH1-mutant or -wildtype astrocytoma patients, Table S5: Function of genes identified in Figure 2, Table S6: Coding mutation frequency in IDH1-mutant or -wildtype oligodendroglioma patients, Table S7: Promoter mutation frequency in IDH1-mutant or -wildtype oligodendroglioma patients, Table S8: Terminator mutation frequency in IDH1-mutant or -wildtype oligodendroglioma patients, Table S9: Intronic mutation frequency in IDH1-mutant or -wildtype oligodendroglioma patients, Table S10: CRAVAT analysis of frequently mutated genes in IDH1-mutant astrocytoma, Table S11: CRAVAT analysis of frequently mutated genes in IDH1-wildtype astrocytoma, Table S12: Gene expression levels in IDH1-mutant or -wildtype astrocytoma patients.
