# Supplementary material for: A Genome-Wide Profiling of Glioma Patients with an IDH1 Mutation Using the Catalogue of Somatic Mutations in Cancer Database
Source: Cancers (Basel). 2021 Aug 26;13(17):4299. doi: 10.3390/cancers13174299 (PMC8428353; doi:10.3390/cancers13174299)
Supplement: Supplementary file 1 [file cancers-13-04299-s001.zip › IDH1_Supplemental Figures_final.pdf]

*Supplementary Materials*

# A Genome-Wide Profiling of Glioma Patients with an IDH1 Mutation Using the Catalogue of Somatic Mutations in Cancer Database

**Amrit L. Pappula [1], Shayaan Rasheed [2], Golrokh Mirzaei [3], Ruben C. Petreaca [4,5] and Renee A. Bouley [6,*]**

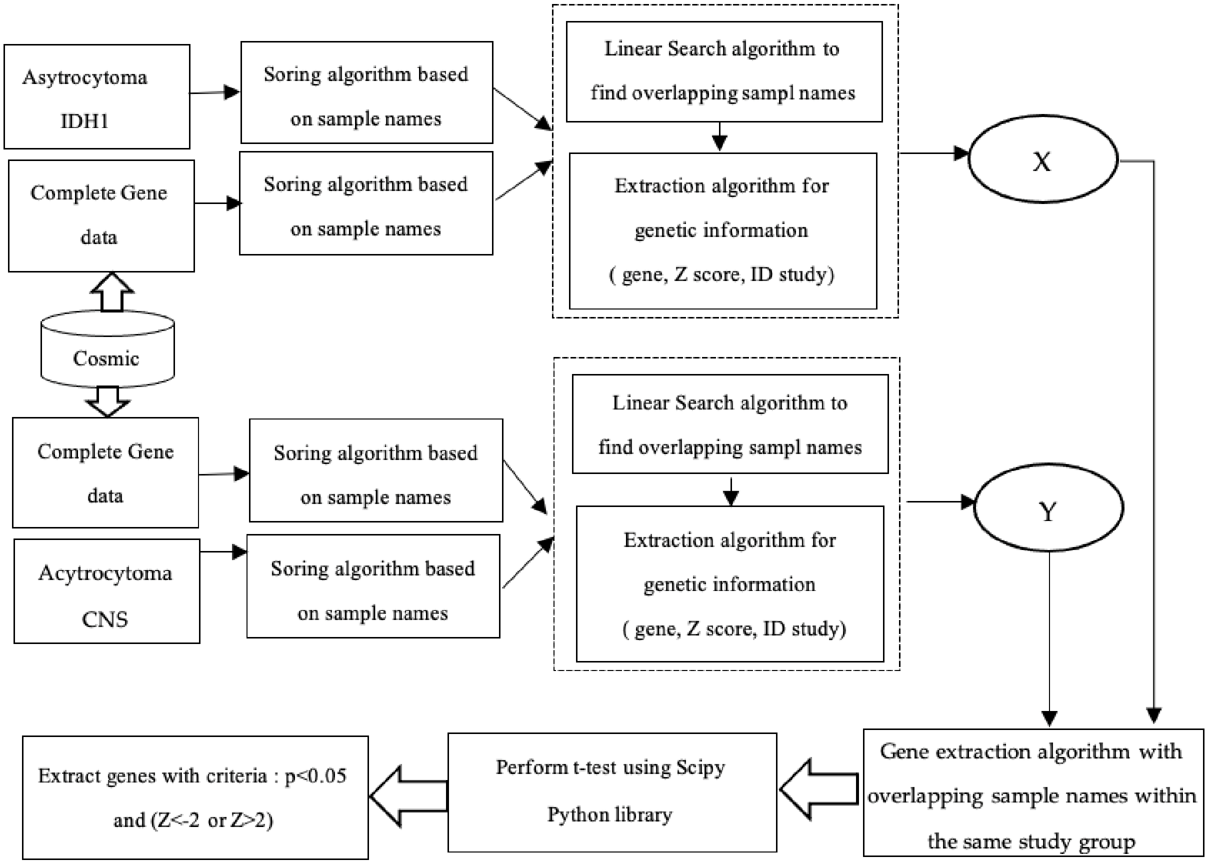


**Figure S1.** The diagram for computational analysis of astrocytoma IDH1-mutant and -wildtype groups.

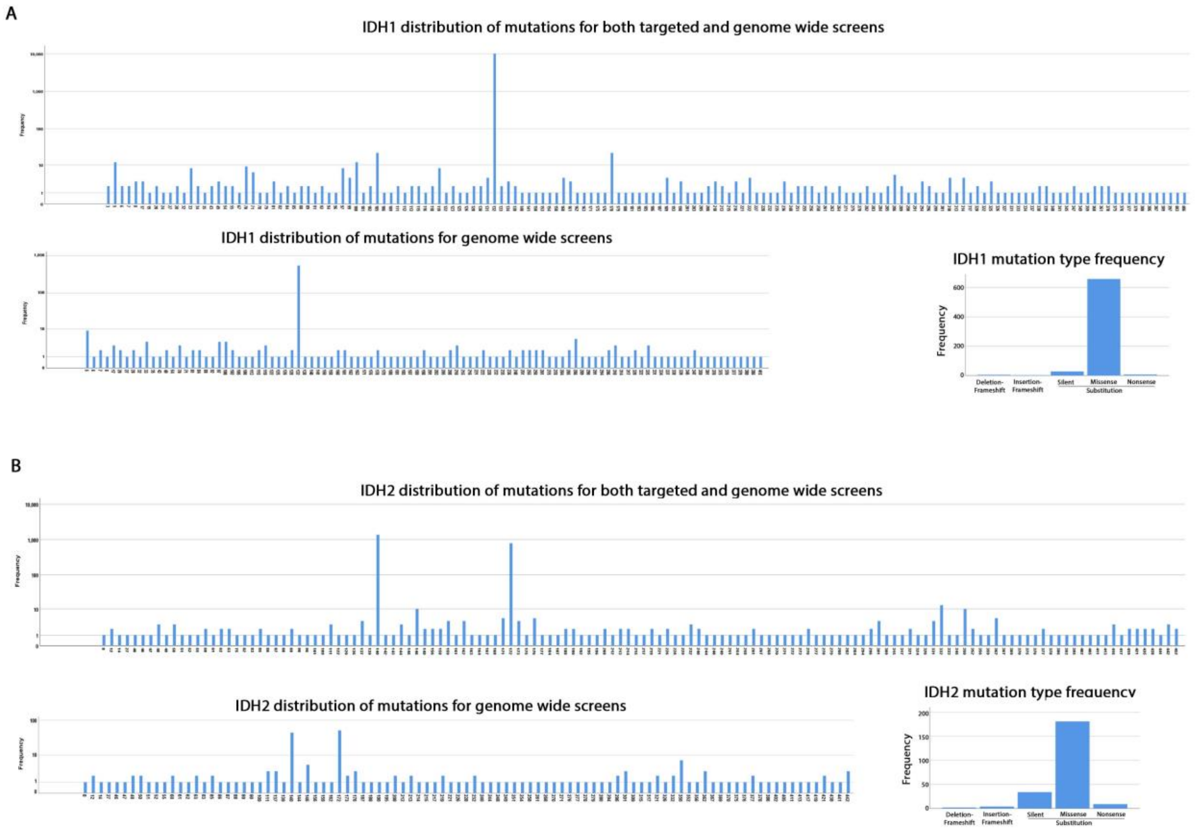


**Figure S2.** IDH1 and IDH2 mutation distribution in all sequenced samples. (**A**) Top graph shows frequency of amino acid mutated in all tissues for IDH1 (targeted and genome wide screens combined). Bottom graph shows frequency for genome-wide screens only. Bottom-right graph shows the frequency of deletion-frameshift, insertion-frameshift, silent, missense, or nonsense mutations. **(B)** Frequency of amino acid mutations in IDH2, following the same layout as described for panel A.

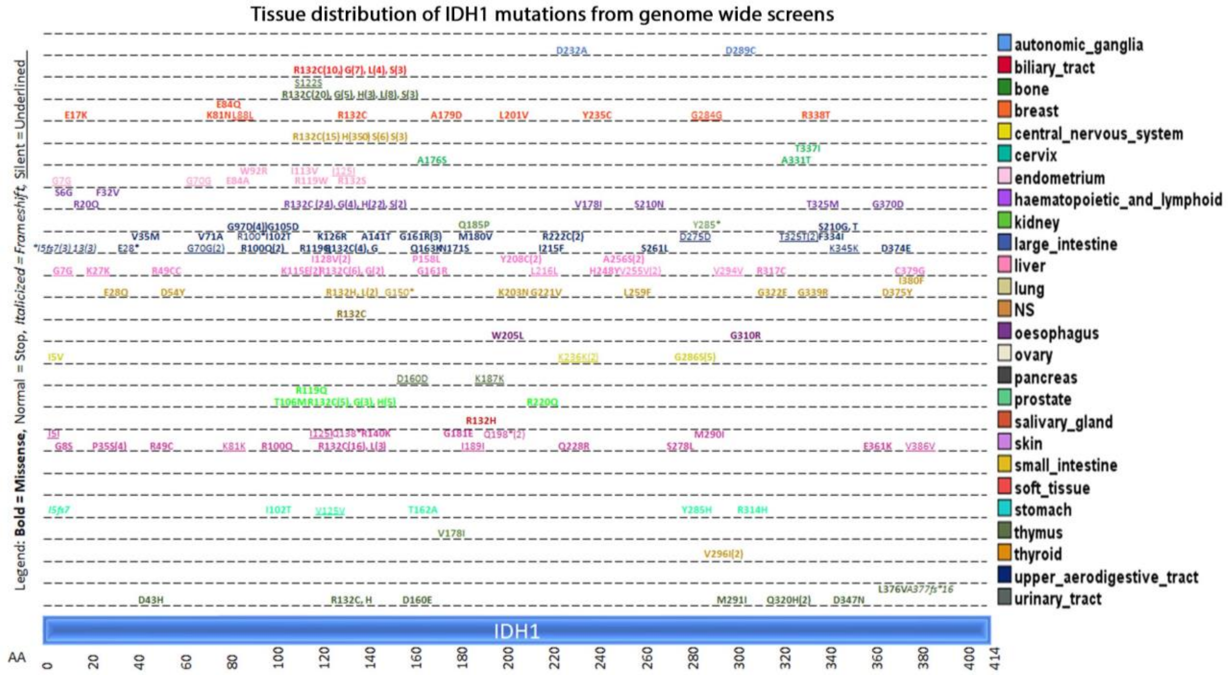


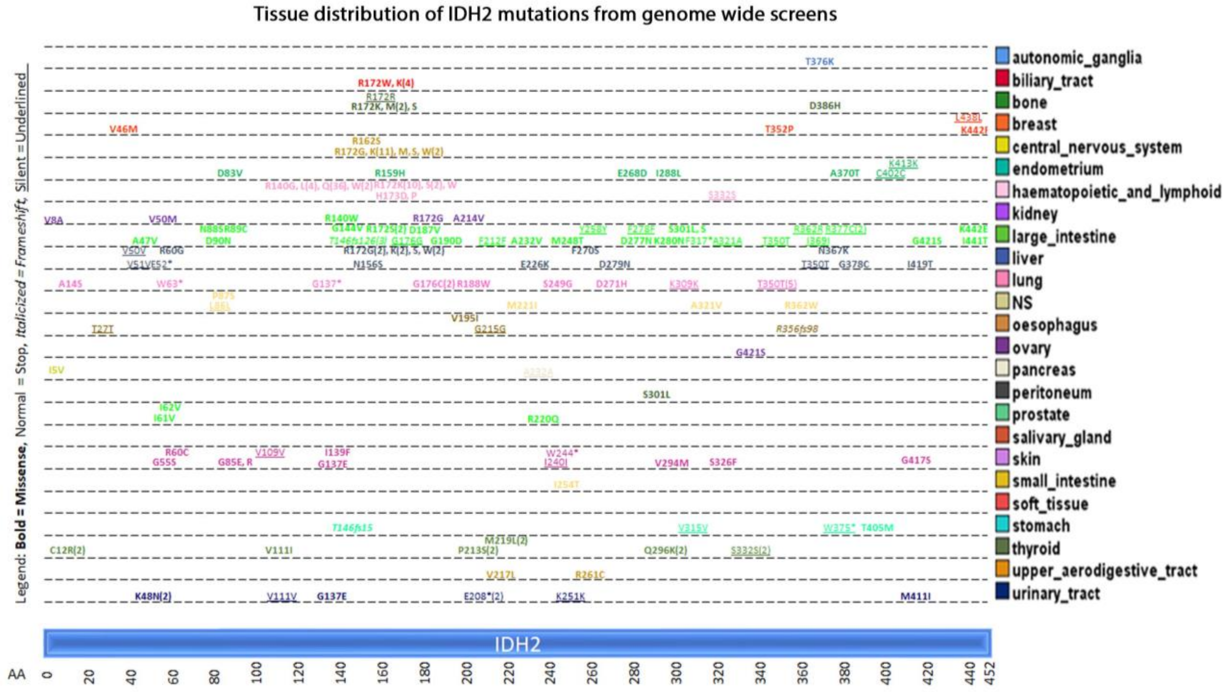


**Figure S3**. IDH1 and IDH2 tissue distribution of mutations in genome wide screens. Maps of all IDH1 and IDH2 mutation locations by tissue for genome wide screens. Shown are all COSMIC reported mutations including silent mutations. Numbers in parenthesis represent the number of times that exact mutation has been reported in that tissue. For example, R132C(10) appeared in ten independent tissue samples. If an amino acid has been independently mutated to different

amino acids this is indicated by several letters separated by comma. For example R132C, G, L(3), S indicates that four different substitutions for R have been reported in six patients, one each to C, G, S and three to L.

A

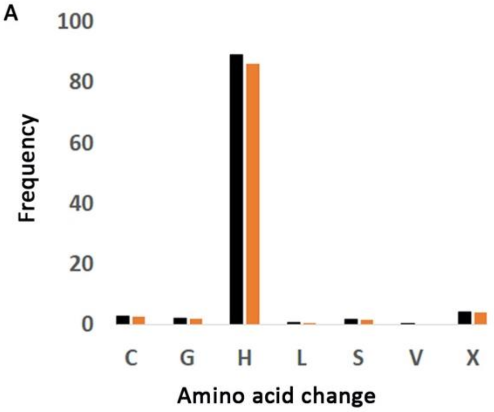


B

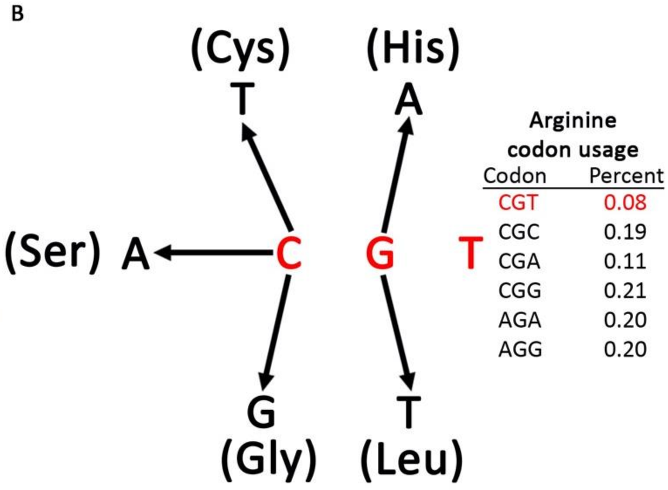


**Arginine codon usage**

| Codon | Percent |
|---|---|
| CGT | 0.08 |
| CGC | 0.19 |
| CGA | 0.11 |
| CGG | 0.21 |
| AGA | 0.20 |
| AGG | 0.20 |

C

**Serine**

| Codon | Percent |
|---|---|
| TCT | 0.18 |
| TCC | 0.22 |
| TCA | 0.15 |
| TCG | 0.06 |
| AGT | 0.15 |
| AGC | 0.24 |

**Cysteine**

| Codon | Percent |
|---|---|
| TGT | 0.45 |
| TGC | 0.55 |

**Glycine**

| Codon | Percent |
|---|---|
| GGT | 0.16 |
| GGC | 0.34 |
| GGA | 0.25 |
| GGG | 0.25 |

**Histidine**

| Codon | Percent |
|---|---|
| CAT | 0.41 |
| CAC | 0.59 |

**Leucine**

| Codon | Percent |
|---|---|
| TTA | 0.07 |
| TTG | 0.13 |
| CTT | 0.13 |
| CTC | 0.20 |
| CTA | 0.07 |
| CTG | 0.41 |

D

| | Cys | Gly | His | Ser | Leu | |
|---|---|---|---|---|---|---|
| | 5.022 | 0.922 | 5.696 | 0.536 | 0.948 | Driver mutations (From Anoosha et al, 2016) |
| Arg | 2.73 | 0.606 | 3.019 | 0.747 | 1.074 | Passenger mutations (From Anoosha et al, 2016) |
| | 2.73 | 2.00 | 88.9 | 1.68 | 0.59 | Percent observed in cancers |

E

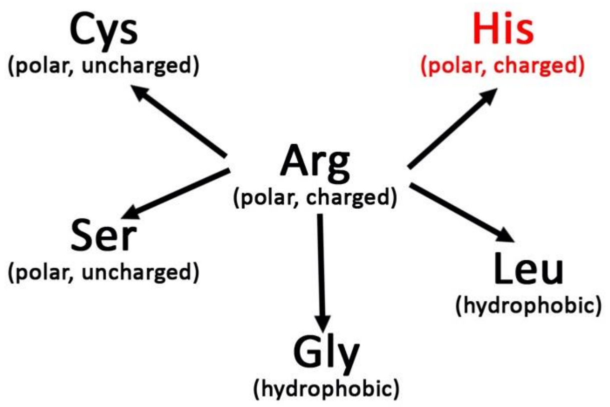

**Figure S4.** Mutation probability of the CGT codon that codes for arginine. **(A)** Frequency of amino acid substitutions for Arg encoded by the CGT codon. **(B)** Possible amino acid mutations possible from single-base substitutions. Percent usage of the different Arg codons. **(C)** Percent usage of corresponding codons resulting from single-base substitutions of CGT. **(D)** Percent of Arg substitutions observed in cancer that are either driver or passenger mutations. **(E)** Chemical and electrostatic properties of amino acid substitutions possible from the Arg CGT codon.

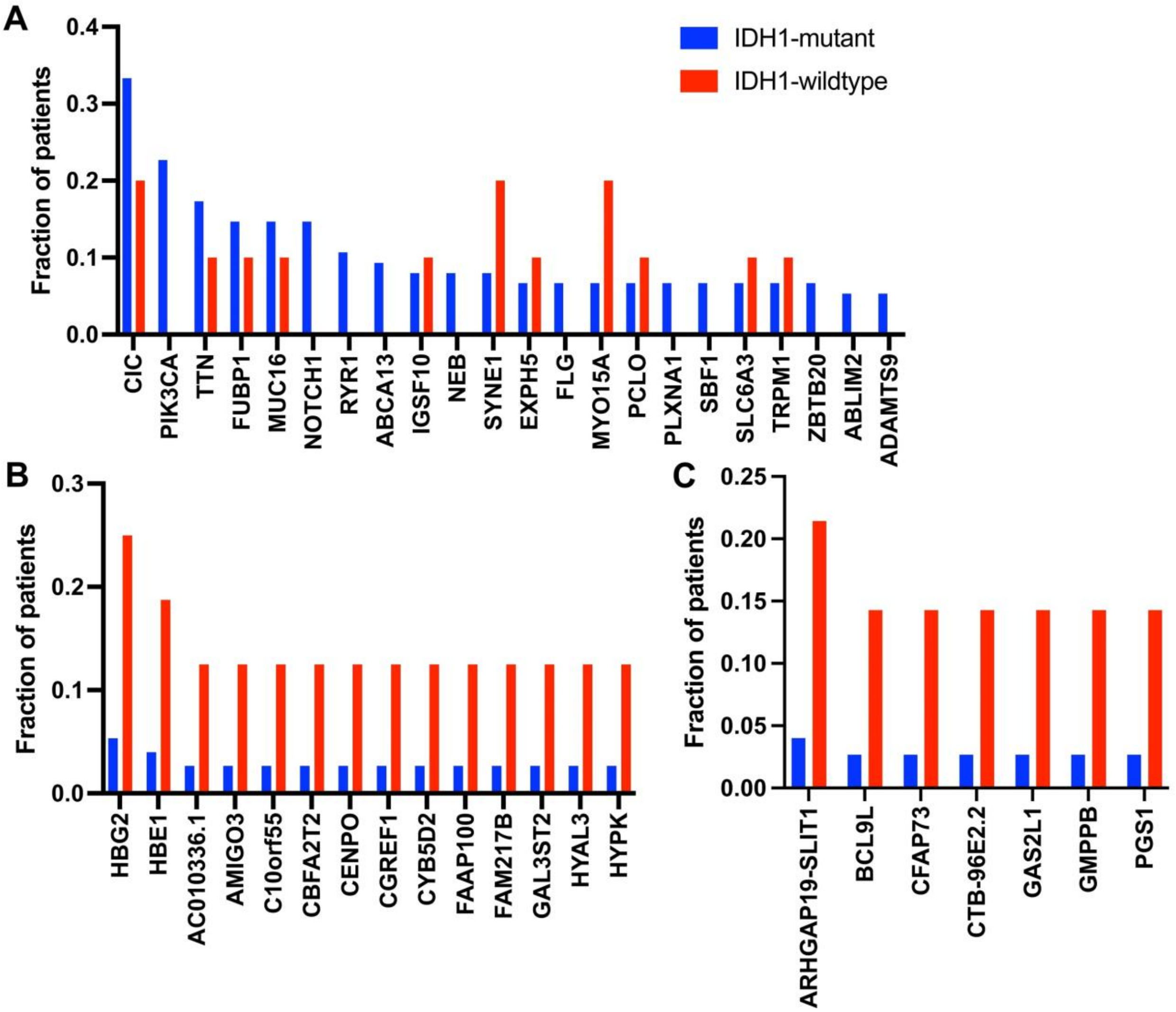


**Figure S5.** Frequency of mutations in IDH1-mutant and -wildtype oligodendroglioma patient samples**.** The number of individual patients displaying a mutation in a given gene was counted and the percentage was calculated from the total number of patients in the dataset. Mutations were split by **(A)** coding, **(B)** promoter, and **(C)** terminator.
